# Initial Mapping of the New York City Wastewater Virome

**DOI:** 10.1128/mSystems.00876-19

**Published:** 2020-06-16

**Authors:** K. Gulino, J. Rahman, M. Badri, J. Morton, R. Bonneau, E. Ghedin

**Affiliations:** a Department of Biology, Center for Genomics and Systems Biology, New York University, New York, New York, USA; b Flatiron Institute, Simons Foundation, New York, New York, USA; c Computer Science Department, Courant Institute, New York University, New York, New York, USA; d Department of Epidemiology, School of Global Public Health, New York University, New York, New York, USA; University of California San Diego

**Keywords:** bacteriophage, metagenomics, virome

## Abstract

Bacteriophages are abundant members of all microbiomes studied to date, influencing microbial communities through interactions with their bacterial hosts. Despite their functional importance and ubiquity, phages have been underexplored in urban environments compared to their bacterial counterparts. We profiled the viral communities in New York City (NYC) wastewater using metagenomic data collected in November 2014 from 14 wastewater treatment plants. We show that phages accounted for the largest viral component of the sewage samples and that specific virus communities were associated with local environmental conditions within boroughs. The vast majority of the virus sequences had no homology matches in public databases, forming an average of 1,700 unique virus clusters (putative genera). These new clusters contribute to elucidating the overwhelming proportion of data that frequently goes unidentified in viral metagenomic studies. We assigned potential hosts to these phages, which appear to infect a wide range of bacterial genera, often outside their presumed host. We determined that infection networks form a modular-nested pattern, indicating that phages include a range of host specificities, from generalists to specialists, with most interactions organized into distinct groups. We identified genes in viral contigs involved in carbon and sulfur cycling, suggesting functional importance of viruses in circulating pathways and gene functions in the wastewater environment. In addition, we identified virophage genes as well as a nearly complete novel virophage genome. These findings provide an understanding of phage abundance and diversity in NYC wastewater, previously uncharacterized, and further examine geographic patterns of phage-host association in urban environments.

**IMPORTANCE** Wastewater is a rich source of microbial life and contains bacteria, viruses, and other microbes found in human waste as well as environmental runoff sources. As part of an effort to characterize the New York City wastewater metagenome, we profiled the viral community of sewage samples across all five boroughs of NYC and found that local sampling sites have unique sets of viruses. We focused on bacteriophages, or viruses of bacteria, to understand how they may influence the microbial ecology of this system. We identified several new clusters of phages and successfully associated them with bacterial hosts, providing insight into virus-host interactions in urban wastewater. This study provides a first look into the viral communities present across the wastewater system in NYC and points to their functional importance in this environment.

## Abstract (precis)

Wastewater is a rich source of microbial life and contains bacteria, viruses, and other microbes found in human waste as well as environmental runoff sources. As part of an effort to characterize the New York City wastewater metagenome, we profiled the viral community of sewage samples across all five boroughs of NYC and found that local sampling sites have unique sets of viruses. We focused on bacteriophages, or viruses of bacteria, to understand how they may influence the microbial ecology of this system. We identified several new clusters of phages and successfully associated them with bacterial hosts, providing insight into virus-host interactions in urban wastewater. This study provides a first look into the viral communities present across the wastewater system in NYC and points to their functional importance in this environment.

## INTRODUCTION

Wastewater treatment systems are responsible for transporting raw sewage which contains a rich source of microbes including bacteria, archaea, fungi, protists, and viruses. In addition to transporting human waste, sewage systems can be combined with drainage systems to transport runoff and stormwater, increasing the overall microbial diversity. Previous research on wastewater influent demonstrates that the bacterial communities in raw sewage can serve as indicators of the human population in the surrounding areas, providing a valuable resource to understand population-level traits and health (1–3).

Viruses and, in particular, bacteriophages are also major components of raw sewage due to the high concentrations of nutrients and biomass present in the system. In fact, wastewater systems were shown to have concentrations of 10^8^ virus particles per microliter, which is 10 to 1000 times higher than any other aquatic environment examined and about 10-fold higher than the estimated concentration of bacterial cells (4–6). A proportion of viruses detected in wastewater systems are eukaryotic, and some may cause human infections, such as human adenoviruses, enteroviruses, and polyomaviruses (7, 8). However, the majority of viruses detected have mostly been bacteriophages (7, 8). In addition to being abundant, bacteriophages impact microbial ecology through their interactions with their hosts. They can influence bacterial communities directly by infection, by shuttling genes through horizontal gene transfer, and by providing potential benefits during prophage integration such as virulence and metabolic genes. Bacteriophages also contribute to nutrient cycling and the release of organic matter in the environment (9–11).

Despite the abundance and functional importance of viruses on microbial ecosystems, few broad-scale metagenomic studies have focused on their presence in raw sewage. Here, we profiled the virus communities present in the sewage system across the 5 boroughs of New York City (NYC), building upon previous work that characterized protists and bacteria in this environment (3, 12). The NYC sewage system includes over 7,000 miles of pipes that flow wastewater into 14 treatment plants spanning the 5 boroughs. We used this metagenomic sequence data to identify and functionally profile viruses in wastewater (12). This type of data allows for viral discovery as well as the study of viral dynamics. For example, recent metagenomic analyses reported on the dynamics of virophages and giant viruses in aquatic systems, increasing our understanding of these viruses within this ecological niche (13). With these data we were able to simultaneously identify viruses and their hosts, better understand how they are related to each other, and determine how viral functional profiles differ across samples and boroughs, thus expanding our knowledge of phage dynamics in wastewater.

## RESULTS

### NYC wastewater virus community is dominated by bacteriophages.

**FIG 1:**
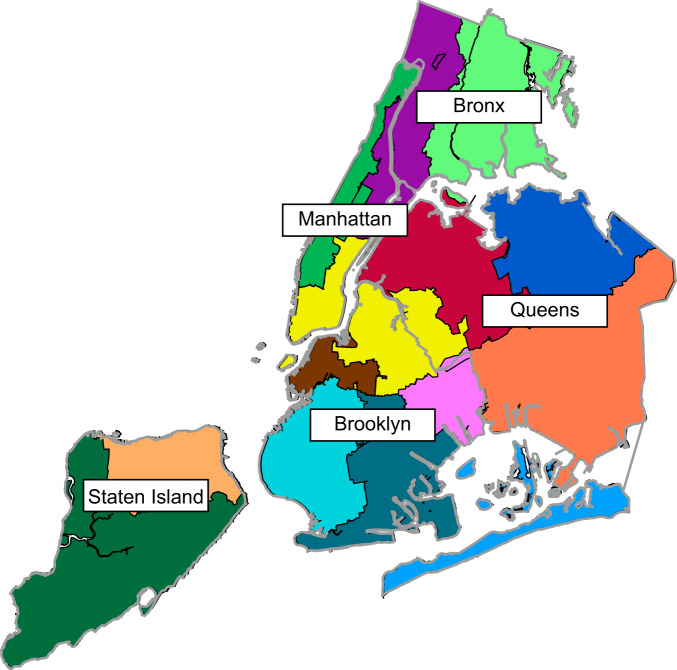
Map of NYC sewage system and sampling sites. Fourteen wastewater treatment catchment areas spread across the 5 boroughs of NYC. Each color represents the catchment area for the wastewater treatment center. Borough boundaries are outlined in gray. Staten Island = dark green, peach; Manhattan = green, yellow, purple; Bronx = purple, light green; Queens = royal blue, orange, yellow, red, light blue; Brooklyn = yellow, brown, aqua, teal, pink. The location data were obtained from https://openseweratlas.tumblr.com/data.

The microbial composition of wastewater in NYC (protists, bacteria) has recently been analyzed using a combination of 18S rRNA and 16S rRNA gene sequencing, and shotgun metagenomics, but viruses have so far been unexplored (3, 12). Here, we used this previously generated shotgun metagenomic data to profile the viral composition of wastewater in NYC. We identified and characterized viruses by analyzing the virus component of metagenomic data from 16 wastewater samples collected in November 2014 across all 5 boroughs of NYC (12) (Fig. 1). On average, there were 10,751,683 total paired-end reads per sample, with 98.2% (10,557,807/sample) of the reads remaining after quality filtering. These sequencing reads were then analyzed for viral signatures, as described below.

To first profile the overall virus taxonomy of this data set, we used VirMAP, a tool developed to merge both nucleotide and protein information to classify viral sequences, while excluding bacterial and eukaryotic sequences (14). This approach allowed us to classify 6,993,448 reads as viral, representing 4.1% of the combined data set of 16 samples. A total of 806 virus taxa were identified. There was an average of 437,090 viral reads per sample with, on average, 166 virus taxa identified per sample (range: 78 to 480). These included eukaryotic viruses and bacteriophages, with a clear dominance of bacteriophage sequences across the data set (eukaryotic viral reads: 431; bacteriophage reads: 6,993,017) (see Data Set S1, Sheet 1, in the supplemental material). However, for the majority of the viral reads (∼90%), taxonomic assignments could not be made beyond “Virus” (taxId = 10239).

10.1128/mSystems.00876-19.10 DATA SET S1 Sheet 1: VirMap viral taxonomy output. Sheet 2: Number of reads and contig statistics per sample. Sheet 3: Phage-host associations by bacterial genera. Sheet 4: Viral cluster and bacterial genera adjacency matrix. Download Data Set S1, XLSX file, 0.1 MB. Copyright © 2020 Gulino et al. 2020 Gulino et al. This content is distributed under the terms of the Creative Commons Attribution 4.0 International license.

**FIG 2:**
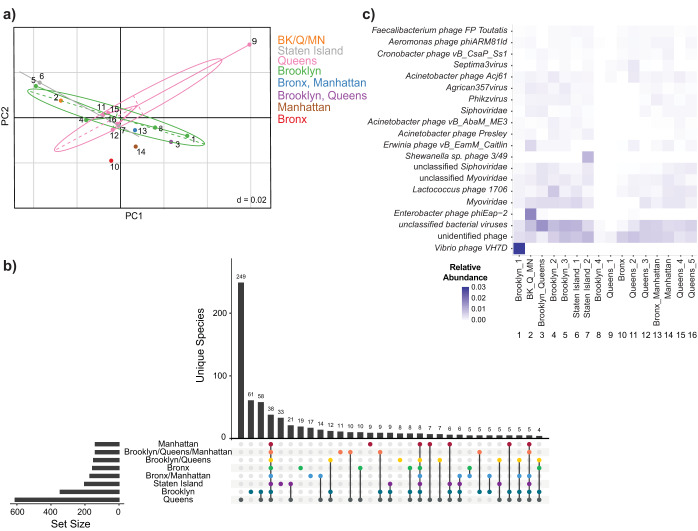
Viral taxonomy and diversity. (a) A PCoA (principal-coordinate analysis) ordination was performed to visualize the viral community matrix across all 16 samples based on the Bray-Curtis dissimilarity measurement. Each color represents the site from which the sample was collected. Samples are numbered and labeled according to collection site. (b) UpSet plot visualizing the intersecting sets of viral taxa at different sewage sampling locations. Each sampling location was defined as a set, resulting in a total of 8 sets (Manhattan = 1 sample, Brooklyn/Queens/Manhattan = 1 sample, Brooklyn/Queens = 1 sample, Bronx = 1 sample, Bronx/Manhattan = 1 sample, Staten Island = 2 samples, Brooklyn = 4 samples, Queens = 5 samples). The “set size” bars over the sets represent the total number of viral taxa present in that set. Dots with interconnecting vertical black lines represent the intersections, where filled and colored dots represent sets that are within the intersection and unfilled light gray dots represent sets that are not part of the intersection. The bars above represent the number of viral taxa within the intersection. (c) Heatmap representing the relative abundance of the top 20 virus taxa (below the “Virus” category), identified by VirMap in each sample. Darker shades of purple indicate higher relative abundance of that virus taxa.

To compare species diversity within each sample and assess how diversity compared across locations, we used measures of alpha and beta diversity, respectively. When calculating Gini-Simpson’s Index (1-Simpson’s Index), where values range from 0 to 1 according to increasing diversity, the average value across these samples was 0.90, though evenness scores, measured by Pielou’s J, were low (Table S1). We observed high alpha diversity in each sample but with uneven species distribution, indicating that only a few species dominate each sample. We next calculated the beta diversity using the Bray-Curtis dissimilarity measurement to measure diversity between samples. The average beta-diversity score was 0.04, indicating that the samples have very similar species composition. Location did not appear to influence diversity among samples (ANOVA, *P* value = 0.615) (Fig. 2a). For example, the Brooklyn samples (green) share a low similarity score as they do not cluster together based on composition and diversity. Overall, virus taxon diversity is not specific to the borough but rather to the sample site, i.e., neighborhoods covered in the catchment area, reflecting the variety of urban ecosystems.

10.1128/mSystems.00876-19.7 TABLE S1 Alpha diversity measurements. Download Table S1, PDF file, 0.2 MB. Copyright © 2020 Gulino et al. 2020 Gulino et al. This content is distributed under the terms of the Creative Commons Attribution 4.0 International license.

To identify patterns of viruses present within and between boroughs, we calculated the intersections of each set of virus taxa by sampling location (Fig. 2b). There were 38 viruses identified in all sampling locations, which we termed the core virome. It included, among others, phages that infect Faecalibacterium prausnitzii, a resident of the human gastrointestinal tract (15); *Lactococcal phage 1706*, known to infect bacteria in the human gut (16); and *Salmonella-* and *Enterobacter*-infecting phages. Queens had the highest number of unique phages (*n* = 249), i.e., phages not shared by any other combination of locations. Brooklyn and Queens shared the highest number of unique virus taxa between sample locations, indicating that these two boroughs may share similar ecological niches that influence virus diversity. These boroughs are distinctly less urban in regard to population density compared to Manhattan.

To characterize in more detail the abundant phages across the data set, we queried for the top 20 most abundant taxonomic classifications (following the nonspecific “Virus” category). These included *Siphoviridae* phages, such as *Lactococcus phage 1706* and *Enterobacter phage phiEap-2*; *Myoviridae* phages, such as *Vibrio phage VH7D* and *Shewanella* sp. *phage 3/49*; and *Podoviridae* phages, including Acinetobacter *phage Presley* and *Cronobacter phage vB_CsaP_Ss1* (Fig. 2c). Identified eukaryotic viruses were mainly from the *Phycodnaviridae* and *Adenoviridae* families, such as *Ostreococcus lucimarinus virus 7* and *Human adenovirus 12*, respectively (Data Set S1, Sheet 1). Viruses in the *Phycodnaviridae* family infect marine and freshwater eukaryotic algae while viruses in the *Adenoviridae* family have a broad range of vertebrate hosts including humans, cats, and dogs.

### The sewage virome contains largely unexplored sequence space.

To further characterize the virome, we ascertained features such as virus environmental sources, bacterial hosts, and functional potential. We expanded beyond a strictly reference-based approach and assembled all sequencing reads from each sample into contigs, which allowed us to do more in-depth analyses to uncover potential viral sequences that often go unexplored in metagenomic studies (Data Set S1, Sheet 2). The contigs generated were used as input for VirSorter (17), which predicts viral contigs based on the presence of virus “hallmark” genes and other virus-specific parameters. This method primarily facilitates the identification of unknown or previously unidentified phages. VirSorter classifies the putative viral contigs into three categories based on confidence, with Category 1 containing contigs with the most support and Category 3 containing those with the least. VirSorter predicted a total of 4,881 viral contigs across all samples (2.2% of reads mapped back). There was a total of 1,095 contigs in Category 1, 3,683 contigs in Category 2, and only 103 contigs in Category 3 (Fig. S1).

10.1128/mSystems.00876-19.1 FIG S1 Proportion of VirSorter categories. Viral contigs identified by VirSorter are distributed across 3 categories. Category 1 (red) represents the highest confidence, Category 2 (yellow) represents high confidence, and Category 3 (blue) represents questionable confidence. Total viral contig count is listed on the *y* axis. BK, Brooklyn; Q, Queens; MN, Manhattan; BX, Bronx. Download FIG S1, PDF file, 0.4 MB. Copyright © 2020 Gulino et al. 2020 Gulino et al. This content is distributed under the terms of the Creative Commons Attribution 4.0 International license.

To understand the genetic relatedness of the viral contigs, we applied a gene content-based network analysis (18) to group predicted contigs based on their gene sequences into virus clusters (VCs), where nodes are genomes or contigs, and edges between nodes represent gene content similarities. In this framework, viruses sharing a high number of genes are organized into VCs that represent approximate virus genera, as defined by the International Committee of Taxonomy of Viruses (ICTV). We represented the relationships among the predicted viral contigs with known bacterial and archaeal viruses from RefSeq as a weighted network for each sample.

**FIG 3:**
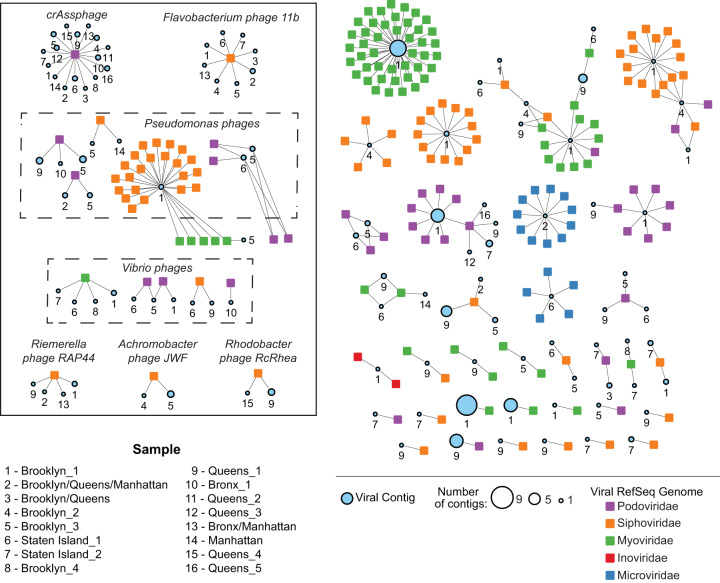
Virus clusters associated with viral RefSeq genomes. Virus clusters (VCs) generated from a gene content-based network analysis that were grouped with RefSeq genomes. Each colored square represents a RefSeq genome, and each circle represents a sewage sampling site. The size of the circles indicates how many viral contigs are within the VC, and numbers under the circles indicate the sampling site.

An average of 2,113 VCs were predicted for each sample. Only 4% of the viral contigs across all samples clustered with RefSeq virus genomes; these contigs can be assumed to be in the same virus genera as the corresponding RefSeq genome (Fig. 3). Each sample had at least one viral contig that was grouped into a VC with the prototypical crAssphage genome (Fig. 3, box), a recently identified ubiquitous phage found in the human intestinal tract (19, 20). *Flavobacterium phage 11b*, which is typically found in the aquatic environment, clustered with viral contigs in half of the samples (Fig. 3, box). Additionally, viral contigs in 7/16 samples clustered with *Vibrio* phages and viral contigs in 8/16 samples clustered with *Pseudomonas* phages (Fig. 3, box). Samples 1, 2, 9, and 13 from Brooklyn_1, Brooklyn/Queens/Manhattan, Queens_1, and Bronx/Manhattan, respectively, had contigs that fell into a cluster with *Riemerella phage RAP*44. This phage infects Riemerella anatipestifer, which causes infection in young ducks and geese (21). Additionally, some patterns were sample or borough specific. For example, two Queens samples (9 and 15) both had viral contigs that clustered with *Rhodobacter phage RcRhea* known to infect the photosynthetic bacterium Rhodobacter capsulatus (22). Two samples from Brooklyn (4 and 5) clustered with *Achromobacter phage JWF*, which was recently isolated from sewage and found to infect the bacterium Achromobacter xylosoxidans, an emerging nosocomial pathogen typically found in wet environments (23).

However, the majority of contigs (96%) did not belong to VCs with RefSeq virus genomes but instead clustered together into novel VCs. There was an average of 1,754 VCs that contained only viral contigs and no RefSeq genomes.

### Identification of novel virophages in wastewater.

In the previous VirMAP analysis, we identified a small number of reads (*n* = 10) belonging to the giant virus family *Mimiviridae* (Data Set S1, Sheet 1). Since this data set was highly diverse and rich in uncharacterized viral sequences, we next searched for evidence of virophage genomes. Virophages are small viruses that use the replication machinery of giant viruses to infect eukaryotic cells including algae and amoebae (24–26). By coopting the giant virus replication machinery, virophages have a negative effect on giant virus replication (26). As such, virophages are responsible for reducing the mortality rate of the eukaryotic cells and could result in algal blooms (26, 27). To date, only two types of virophages have been isolated in culture. However, using metagenomics, 57 partial and complete virophage genomes have been identified (26). We first looked for the major capsid protein (MCP) because it is a conserved virophage marker gene. This search revealed 48 MCP-containing contigs, nearly doubling the total number of virophage MCPs previously identified. The MCPs were identified in 11 out of the 16 samples, and these were found in every borough except Manhattan. The sample with the highest number of contigs with an MCP protein was Queens_1 (14 contigs). All MCPs matched the Zamilon/Sputnik MCP hidden Markov model (HMM) profile.

**FIG 4:**
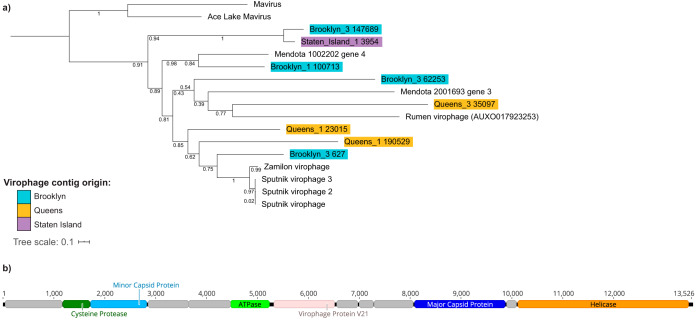
Maximum-likelihood virophage major capsid protein (MCP) tree. (a) MCPs from known virophages and from contigs identified in this study were aligned, and a maximum likelihood tree was constructed. The tree was rooted using the *Mavirus* virophage. Contigs from this study are highlighted in a color corresponding to borough. (b) Genome organization of Brooklyn_3 627, which contains a cysteine protease, minor capsid protein, ATPase, major capsid protein, helicase, and other virophage and predicted genes.

We constructed a maximum-likelihood phylogenetic tree of the identified full and nearly complete MCPs in this data set along with previously published MCP sequences from GenBank and RefSeq (Fig. 4). The contig Brooklyn_3_627 contained a complete MCP protein and clusters most closely with the Sputnik/Zamilon MCP proteins, though it forms a distinct branch (Fig. 4a). The other virophage contigs cluster with freshwater virophages such as Mendota (13) and a virophage identified in sheep rumen. Additionally, the Brooklyn_3_147689 and Staten_Island_1_3954 contigs cluster most closely with one another.

We next determined if these virophage contigs also contained three other core virophage proteins: a minor capsid, a cysteine protease, and a DNA-packaging protein. Brooklyn_3_627 contained these additional core proteins (Fig. 4b). Staten_Island_1_3954 contained all other core genes except the minor capsid. These core genes are found in all virophage genomes analyzed thus far and indicate that these newly discovered partial and nearly complete genomes contain the essential virophage genes.

### Sewage contains viruses from different environmental sources.

**FIG 5:**
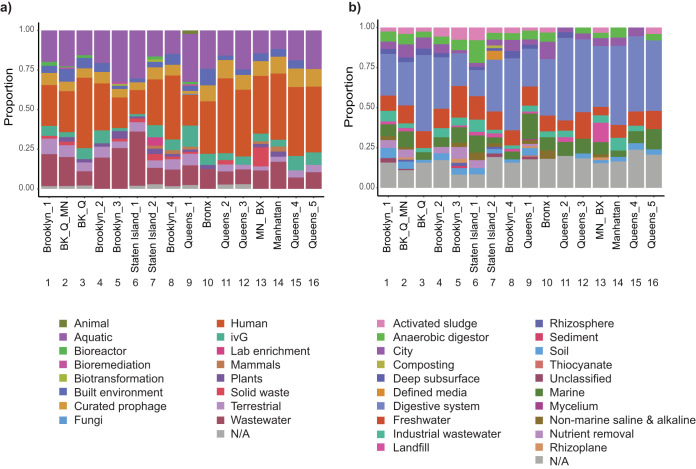
Environmental sources of viral contigs. (a) Proportion of viral contigs that mapped back to IMG/VR contigs by environment. Each color represents a different environment. Samples are labeled by number and by sampling site. (b) Proportion of viral contigs that mapped back to IMG/VR contigs by habitat. IMG/VR habitats are more specific than environments. Colors represent habitat. BK, Brooklyn; Q, Queens; MN, Manhattan; BX, Bronx.

Viruses present in sewage systems may come from a variety of sources in addition to the human body. NYC has a combined sewage system, where runoff water, rainwater, and waste enter into the wastewater system. To identify the potential environmental sources of the viral contigs, VirSorter contigs from the higher-confidence categories (1 and 2) were compared to the Integrated Microbial Genome/Virus (IMG/VR) database (28), which contains viral metagenomic data sets from several different sources, including wastewater. The top three sources for these matches were samples originating from humans (1,511 contigs), aquatic environments (1,158 contigs), and wastewater (823 contigs) (Fig. 5a). Sample 9, from Queens, was the only sample with contigs matching sources from animals. Only 5 of the samples had matches to a bioreactor source, and Sample 13 collected from Manhattan/Bronx had the highest abundance of contigs matching solid waste sources. These environments can be further separated into specific categories corresponding to their sources. For example, we identified 1,598 contigs that have sources in the human digestive system (Fig. 5b). The aquatic environment, when separated into different ecosystems, showed 577 contigs that belonged to freshwater and 448 contigs that belonged to the marine environment (Fig. 5b). Twelve of the 16 samples contained matches to sources originating from activated sludge, a common component of the wastewater treatment process. Additionally, Sample 7 from Staten Island was the only sample with hits to composting environments. This sample also had matches to defined media, indicating that some of the viral contigs in this sample may be similar to cultured phages. Overall, these data show that the viral contigs originated from different environmental sources.

### Viral contigs contain a broad range of functional and structural genes.

Phages contain a set of genes that are used to infect hosts, replicate their genomes, and produce new progeny. In addition to these genes, phage genomes may also carry additional genes that can impact their bacterial host, aiding in overall survival by providing metabolic or fitness benefits (29–31). To probe the functional potential of the viral contigs in this data set, we first annotated the predicted open reading frames using the UniRef50 database, which contains clustered sets of protein sequences, and mapped the annotations to corresponding Gene Ontology (GO) terms (32) (Fig. S2). Top GO terms across the samples were relevant to phages and included biological processes such as DNA integration and replication, cellular components such as viral capsid, and molecular functions such as ATP binding and endonuclease activity.

10.1128/mSystems.00876-19.2 FIG S2 Viral contig Gene Ontology (GO) terms. Bubble plot displaying the relative abundance (square root) of the identified GO terms. The size and shade of the circle indicate relative abundance. Terms highlighted in blue are those of interest noted in the text. BK, Brooklyn; Q, Queens; MN, Manhattan; BX, Bronx. Download FIG S2, PDF file, 1.3 MB. Copyright © 2020 Gulino et al. 2020 Gulino et al. This content is distributed under the terms of the Creative Commons Attribution 4.0 International license.

**FIG 6:**
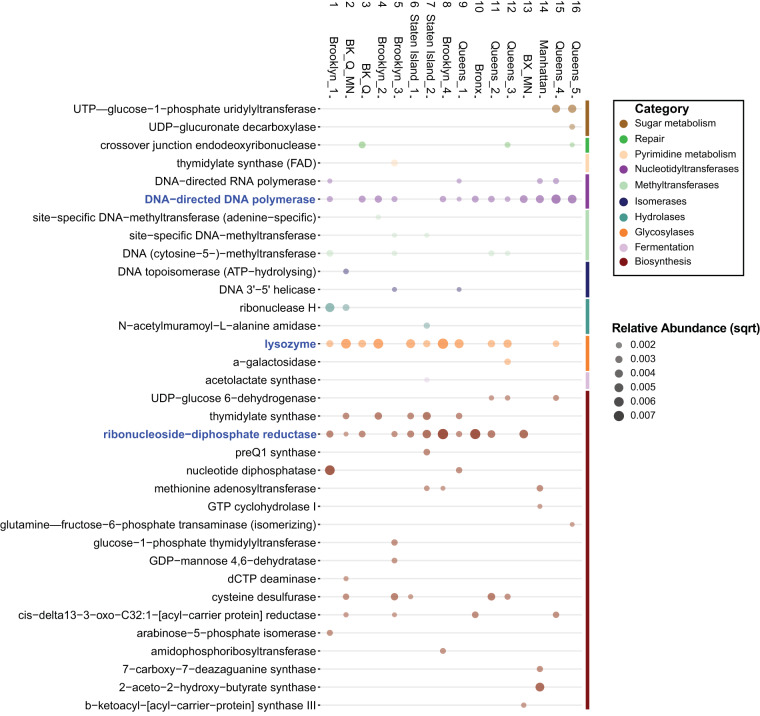
Relative abundance of metabolic pathway genes in viral contigs. Bubble chart representing the relative abundance (square root) of metabolic enzymes present on viral contigs. Enzymes were identified using the MetaCyc enzymatic reaction database. The size and shade of the bubble are proportional to the relative abundance of the gene. Samples are labeled by number and by sampling site. Enzymes highlighted in blue are those of interest noted in the text. BK, Brooklyn; Q, Queens; MN, Manhattan; BX, Bronx.

We also mapped the UniRef50 matches to the MetaCyc enzymatic reaction database (32) to determine the metabolic potential of the phages in the sewage system (Fig. 6). Three enzymes—DNA-directed DNA polymerase, lysozyme, and ribonucleoside-diphosphate reductase—were present in at least 11 of the 16 samples. Some enzymes were specific to only a few samples. For example, nucleotide diphosphatase was present in only 2 samples (Brooklyn_1 and Queens_1) and UTP–glucose-1-phosphate uridylyltransferase, an enzyme involved in carbohydrate metabolism, was present in only 2 samples from Queens (Queens_4 and Queens_5).

Furthermore, we identified a total of 8,419 protein families (Pfams) by searching against the Pfam database (33, 34). The majority of the samples, regardless of location, clustered together based on the presence or absence of the Pfam domains, which points toward a subset of protein families that are universally present or absent across the samples (Fig. S3a). Outliers consisted of samples from Queens, Brooklyn, and Staten Island as well as the sample collected from Brooklyn/Queens/Manhattan (Fig. S3a). To more closely examine the protein domains for evidence of auxiliary metabolic genes (AMGs), we removed virus- and phage-associated protein families, resulting in 8,240 protein families. AMGs are found in bacteriophage genomes but are derived from bacterial cells. They can support host metabolism during infection through processes such as photosynthesis, carbon metabolism, and nucleic acid synthesis. Using hierarchical clustering, the samples clustered into 3 groups. The Queens_1 sample (Sample 9) formed its own group, while the other samples were split between the remaining 2 clusters (ANOVA, *P* value < 0.001). There were several protein families present at a higher relative abundance in the Queens_1 sample compared to the other 2 clusters (Fig. S3b). For example, thioredoxin had a higher relative abundance in Queens_1 compared to all other locations. Additionally, there were several differences between clusters 1 and 2, including rhodanese being more highly abundant in cluster 1 (*P* value < 0.01).

10.1128/mSystems.00876-19.3 FIG S3 Diversity of protein families (Pfams) across samples. (a) Multidimensional scaling plot representing the Pfam matrix across all 16 samples based on the Bray-Curtis dissimilarity measurement. Each color represents the site from which the sample was collected. Samples are numbered and labeled according to collection site. (b) Samples were clustered into 3 groups based on the relative abundance of Pfam domains. Terms highlighted in blue are those of interest noted in the text. BK, Brooklyn; Q, Queens; MN, Manhattan. Download FIG S3, PDF file, 0.6 MB. Copyright © 2020 Gulino et al. 2020 Gulino et al. This content is distributed under the terms of the Creative Commons Attribution 4.0 International license.

A total of 3,248 (39%) of the remaining protein families were universally present across the samples. We computed the core protein families by selecting for those with a relative abundance greater than 0.25% in at least 75% of the samples. In doing so, we identified 31 core protein families that included ABC transporters, which are involved in the shuttling of various substrates (35); SusD/RagB, important for nutrient binding (36); and tetR, which confers bacterial resistance to tetracycline (37) (Table S2).

10.1128/mSystems.00876-19.8 TABLE S2 Core protein families. Download Table S2, PDF file, 0.2 MB. Copyright © 2020 Gulino et al. 2020 Gulino et al. This content is distributed under the terms of the Creative Commons Attribution 4.0 International license.

### Identified prophage sequences are mostly unique.

Some phages can integrate into bacterial genomes, where they are termed prophages. Prophages can influence the fitness and virulence of the bacterial host (31). They can encode auxiliary metabolic genes, as described above, and are responsible for a large proportion of bacterial genetic diversity (30, 38). We identified 140 prophage-associated contigs across the samples using the PHAge Search Tool (PHASTER [39]). Of these, 57% aligned only to themselves following an all-versus-all BLASTN search, whereas 43% aligned to at least one other contig in the data set, suggesting that the majority of prophage sequences in this data set are unique. Of the 140 contigs, we identified six sequences that contained intact prophage genome regions. They spanned from 15 kb to 67.8 kb in length, with an average length of 36.5 kb. The regions around five of the six prophage sequences could be assigned a specific host and matched different bacterial species including Ketobacter alkanivorans, Moraxella osloensis, Fusobacterium periodonticum, *Sphingobacteriaceae* bacterium, and Bacillus cereus.

### Phage-host interactions are diverse and modular.

Studies carried out in natural environments suggest that environmental conditions influence phage-host range and that phage-host ranges may be broader than originally suspected (40). To determine patterns of phage-host interactions in this urban environment, we identified CRISPR spacers, which originate from infecting virus genomes, and direct repeats, present in the bacterial genome. We identified an average of 20,735 spacers and 1,686 repeats per sample (Table S3). The Brooklyn/Queens/Manhattan sample had the most identified spacers and repeats (38,687 and 2,929, respectively). This could be attributed to having sewage flow from three boroughs, the most out of any of the samples. We also analyzed the proportion of spacers identified in bacteria attributed to various human body sites (41). We show that most samples have a high proportion of spacers identified in *Moraxella* as well as a wide range of gut-associated bacteria (Fig. S4).

10.1128/mSystems.00876-19.4 FIG S4 Spacers identified in bacteria associated with different human body sites. Bubble chart showing the relative proportions of spacers identified in various bacterial genera. Bacterial genera were previously associated with human body sites, which are indicated in different colors (yellow = gut, green = oral, purple = skin, red = vaginal). The size and shade of the circle indicate proportion. BK, Brooklyn; Q, Queens; MN, Manhattan; BX, Bronx. Download FIG S4, PDF file, 0.5 MB. Copyright © 2020 Gulino et al. 2020 Gulino et al. This content is distributed under the terms of the Creative Commons Attribution 4.0 International license.

10.1128/mSystems.00876-19.9 TABLE S3 CRISPR array identification and clustering. Download Table S3, PDF file, 0.5 MB. Copyright © 2020 Gulino et al. 2020 Gulino et al. This content is distributed under the terms of the Creative Commons Attribution 4.0 International license.

**FIG 7:**
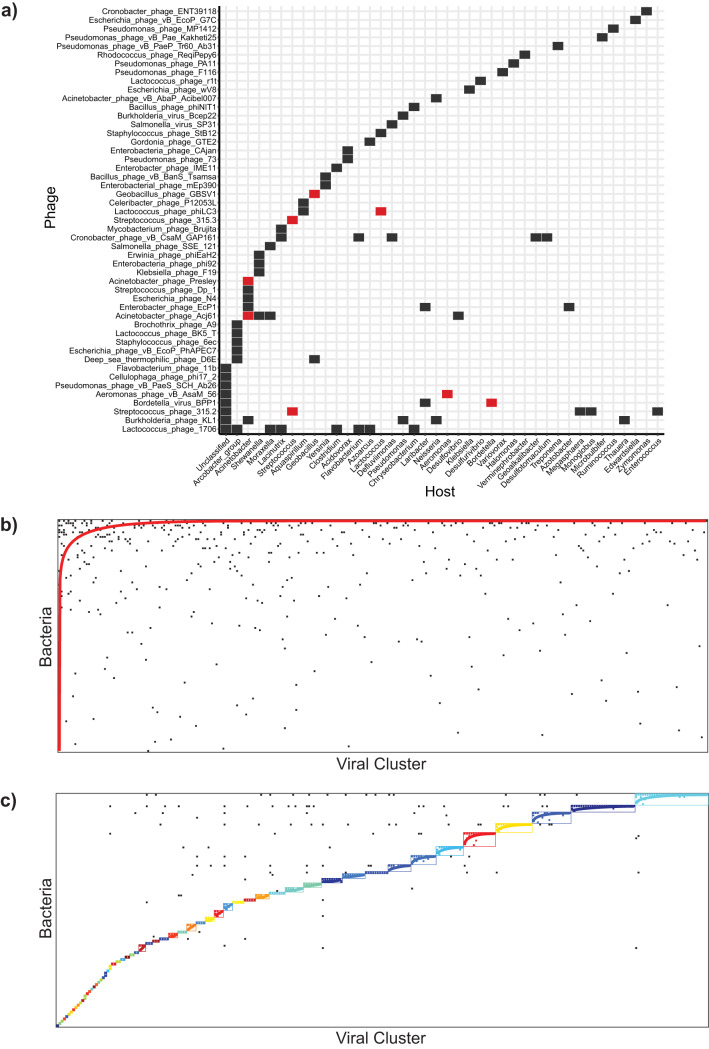
Phage-host interactions by bacterial genera. (a) Phage-host pairs are indicated for the viral contigs that could be assigned taxonomy, resulting in 91 pairs. Bacterial genera are displayed on the *x* axis and viruses on the *y* axis. A box indicates the presence of a CRISPR spacer linking the phage to the host. A red box indicates the phage infects its assigned database host. (b) The phage-bacterium interaction matrix was sorted to maximize nestedness (N_NTC_ = 0.97, NODF = 0.0472). The red curve represents an isocline of perfect nestedness. (c) The phage-bacterium interaction matrix was sorted for modularity. Fifty-eight modules were detected using LP-BRIM (Qb = 0.716). Each color represents a different module, with corresponding isoclines. Black boxes indicate interactions that occur outside the module.

We were able to assign 929 hosts to the viral contigs using the spacer sequences. At the genus level, the highest number of phage-host interactions were with Acinetobacter, *Arcobacter*, and *Moraxella* (Data Set S1, Sheet 3). There were also sample-specific phage-host interactions. For example, only phages from Staten Island were linked to *Geobacillus* hosts, and only phages from Queens were linked to bacteria in the genus *Dialister* (Data Set S1, Sheet 3). We successfully assigned specific phage taxonomy to 91 of the phage-host pairs (Fig. 7a). Of these 91 pairs, only 8 phages were identified to infect their taxonomically assigned host. For example, *Streptococcus phage 315.2* was linked back to *Streptococcus*, and *Geobacillus phage GBSV1* was paired with *Geobacillus*. The phage with the broadest host range was *Lactococcus phage 1706*, which was linked back to eight different bacterial genera, suggesting that this phage may be a generalist in the urban sewage environment. Together, these results demonstrate that phage-host interactions in the urban sewage environment are broad and can span genera.

The ability to assign viral contigs to hosts using CRISPR spacers allowed us to determine the underlying network structure of the phage-host infection patterns we identified. We applied the gene content-based network analysis as described above to the 929 viral contigs that were linked to bacterial hosts. This resulted in 285 VCs which infect a total of 102 different bacterial genera (Data Set S1, Sheet 4). We used this binary adjacency matrix to calculate the nestedness and modularity of the phage-host infection networks. Nested networks represent those in which there is a hierarchy of phages that can infect susceptible hosts. In a nested network, phages with a broad host range can infect all hosts, whereas specialist phages infect only one host. In modular networks, infections take place between phages and bacteria in the same subset, rather than across the different subsets; this type of interaction pattern may indicate distinct clusters of phage-host infections. We observed low values for nestedness (nestedness temperature calculator [N_NTC_] = 0.97, nestedness metric based on overlap and decreasing fill [NODF] = 0.0472), and 58 modules (Barber’s modularity [Qb] = 0.716) (Fig. 7b and c). We also observed high nestedness within some of the individual modules, pointing toward a “nested-modular” infection pattern that has previously been observed in the murine gut and ocean data sets (42). This infection pattern is indicative of a framework where phages span from generalists to specialists with interactions organized into modules (43). We also identified generalist phage VCs that interact with a range of bacterial hosts, often outside their assigned module (Fig. S5).

10.1128/mSystems.00876-19.5 FIG S5 Phage-bacterial modular graph. Tanglegram showing the interactions between 285 virus clusters (VCs) and 102 bacterial genera, organized into 58 modules. Each color represents a distinct module, with black lines representing interactions occurring outside a given module. Download FIG S5, PDF file, 0.9 MB. Copyright © 2020 Gulino et al. 2020 Gulino et al. This content is distributed under the terms of the Creative Commons Attribution 4.0 International license.

### Phage-host co-occurrence across sewage systems.

We aimed to understand patterns of phage-host co-occurrence in the wastewater system since phages can interact only with hosts that are present in their environment and information on host and phage distribution in wastewater systems is lacking. We expanded beyond NYC to also include metagenomic data collected from across the United States, with samples from California, Illinois, Massachusetts, Pennsylvania, and Vermont, to better understand these patterns on a broader geographic scale.

Using a multicity approach, we had enough samples to be able to approximate the conditional probability of observing a specific microbe given that a specific phage was observed with enough statistical power. We generated a network to visualize these probabilities using the core phages previously identified (Fig. S6). We predicted over 200 phage-host relationships. Phages within this network tend to co-occur with bacteria in the same phylum. We also observe that *crAssphage*, a highly abundant phage in the human gut, has a high co-occurrence probability with phages within the *Bacteroidetes* phylum, which are known hosts of *crAssphage*. *Lactococcus phage 1706* has a high probability of co-occurrence with bacteria in the *Campylobacteraceae* family, and specifically with *Arcobacter*, a host we identified in the CRISPR spacer analysis, suggesting this may be conserved across wastewater systems. The hosts it co-occurs with overlap *Human gut gokushovirus*, *Aeromonas virus Aes12*, and *Cronobacter phage vB_CsaP_Ss1*, which have all been found in the human gut, suggesting that they share a similar ecological niche. *Faecalibacterium* phages *FP_oengus* and *FP_Toutatis* co-occur with bacteria in the *Firmicutes* phylum, which are known hosts and may be promoting growth of the phage population. Using wastewater data collected across a broader geographic scale allowed us to more accurately identify these phage-bacterium relationships that are present across all samples. This could also provide clues to understand phage-host interactions in bacteria without CRISPR systems (44, 45).

10.1128/mSystems.00876-19.6 FIG S6 Phage-bacterial co-occurrence probabilities. Network visualization of phage-host co-occurrence probabilities across wastewater data sets from the United States. Phages are represented as triangles, and bacteria are represented as circles. The color of the circle indicates phylum-level taxonomy. Lines connect phages to the 10 bacterial taxa they are most likely to co-occur with. The thickness of the line represents the strength of the co-occurrence probability. Download FIG S6, PDF file, 0.5 MB. Copyright © 2020 Gulino et al. 2020 Gulino et al. This content is distributed under the terms of the Creative Commons Attribution 4.0 International license.

## DISCUSSION

This is the first study to examine viruses in sewage collected from New York City, and it adds valuable information to the previous studies on protist and bacterial diversity in this environment (3, 12). Using metagenomic data, we established that the viral component of sewage in NYC is dominated by bacteriophages that are not currently in databases. By combining reference-based and reference-free approaches, which classified 4.1% and 2.2% of reads, respectively, we assigned viral origin to 6.3% of the sequencing reads of this study. This greatly expands on the proportion of viral reads typically examined in an unenriched, metagenomic sample (which can be as low as 1% [7, 46]). From the analysis of virus clusters, we identified multiple viral contigs—including contigs that clustered with crAssphage, an abundant human fecal bacteriophage that could be considered a useful biomarker of fecal contamination in the sewage system process (20)—and a number of contigs unique to this study, potentially representing new phage genera and demonstrating the novelty of viruses present in NYC sewage. This finding is significant in that it points toward the wide range of viruses yet to be discovered in all environments and is consistent with previous studies examining wastewater viromes (5). This is also similar to findings from global ocean virome data sets where ∼1,000 new virus genera were predicted using this framework (18). The exploration of each new environment will continue to add a significant number of novel viral sequences to current databases (47–49).

We also identified 48 virophage MCPs in NYC wastewater. Virophages are small viruses that infect eukaryotic cells by hijacking the infection cycle of coinfecting giant viruses (24, 25). This relationship leads to the fine-tuning of algal and small eukaryote abundance in their environments (27). Only a few virophages have been isolated and cultured, though previous work has demonstrated that virophages can be identified using metagenomic data (13). We identified several virophage MCPs in NYC wastewater that are diverse in nature and span several environments including freshwater, the digestive system, and potentially a sewage-specific environment. We assembled and characterized a nearly complete virophage genome that is most closely related to the Sputnik and Zamilon virophages. This is the first nearly complete virophage genome found in wastewater, supporting evidence that virophages are widely distributed in the environment (50). Further studies examining virophages and their associations with giant viruses in wastewater data sets would provide a deeper understanding of their role in urban environments.

Previous research suggests that although phages are widely distributed in the environment, there are distinctive groups in specific locations (51, 52). We observed geographic similarities and substantial differences among phage taxa in this data set. The viral profiles we identified were more dependent on local wastewater catchment locations (for example, the different Queens samples) than by borough boundaries, indicating that local inputs into the sewage system may play a role in viral diversity at each location. We identified specific patterns across samples as well. For example, while the “core” virome across all sampled sites consisted of 38 shared viruses, samples from Brooklyn and Queens also had 58 unique viruses only shared between them. This suggests that the sewage inputs in these two boroughs may be highly similar, selecting for the presence of unique viruses. Overall, these findings support the conclusion that there can be enrichment of phage taxa at certain locations (52, 53). The analysis of potential environmental sources of viral contigs show that while we could track human, soil, and wastewater as virus sources across all samples, a few sources were unique to sampling sites. For example, in the Staten Island wastewater treatment plant, a unique source of viral contigs came from composting, which was part of a new NYC initiative piloted in Staten Island in 2013–2014. It is interesting that a new program could impact the ecology of an environment so quickly and clearly. In three of the wastewater treatment plants (Brooklyn/Queens/Manhattan, Brooklyn, and Manhattan/Bronx; samples 2, 5, and 13, respectively), rhizoplane sources, originating from plant roots, could be identified, suggesting that runoff from plant-related locations flowed into the sewage system at these sites. The abundance of non-human-associated environments coincides with similar findings of protist communities in NYC (12) as well as previous studies on bacterial communities in different wastewater data sets (2, 54. Additionally, the identified prophages also spanned a range of environments. The corresponding bacterial species had a range of urban habitats including seawater, laundry facilities, human oral cavities, freshwater, and soil, respectively.

It has been established that bacteriophages influence microbial communities and the environments they inhabit. They do so in numerous ways, such as by putting pressure on hosts to evolve to avoid infection (i.e., arms-race dynamics), by conferring advantages to their hosts through auxiliary metabolic genes, and through nutrient cycling and organic matter release (9–11). In this study, we identified genes involved in carbon, sulfur, and carbohydrate metabolism. For example, thioredoxin, a component of carbon metabolism and an absolute requirement for filamentous phage assembly (55), had a higher relative abundance in one Queens sample compared to all other locations, suggesting that there is a higher abundance of filamentous phage at this location. Rhodanese, a central enzyme involved in sulfur metabolism and important for cyanide detoxification, was also identified as having a higher relative abundance in some samples compared with others. This suggests that phages may play a role in sulfur metabolism at sites where these samples were collected (56). We also identified on viral contigs genes that confer resistance to tetracyclines. Tetracycline resistance genes were also observed in the bacterial communities in the NYC sewage (3).

Phage-host infection patterns can explain underlying evolutionary and ecological processes. We showed that phages are capable of infecting several bacterial species, even spanning across bacterial genera, and that many phages can infect bacteria outside their previously assigned/annotated hosts. This supports the notion that phage-host infections in natural environments are complex and can extend to broader taxonomic ranges, beyond known associations provided in current databases (40). Expanding beyond CRISPR-based phage-host analyses, we used a multicity approach to predict the probability of phages and hosts co-occurring together in sewage systems. This analysis predicted over 200 phage-host relationships, some of which had high co-occurrence probabilities. The relationship between phages and hosts having a high probability of being observed together can indicate a few scenarios. The first is that the phage population may provide a benefit to the host it infects. Second, the presence of the host may allow the growth of the phage population. Third, the phage population preys on a competitor of the bacteria it is connected with. Or, fourth, the phage may be generalist in nature and coexist with multiple potential hosts. The relationships predicted in our network could be considered for exploration in future studies.

We also show that phage-host infection networks are mostly modular, with nestedness within individual modules. This infection pattern is indicative of phages that have evolved to infect a range of bacteria within modular constraints and is similar to findings in the murine gut (43). Modular interaction structures are found to occur where there is high availability of resources and high bacterial diversity (57), both of which are true for wastewater (58). Some phage virus clusters, i.e., VCs, were associated with bacteria outside their assigned module, indicating that they may have evolved to infect a broader range of hosts based on host availability or other environmental parameters, such as temperature, nutrient availability, and host susceptibility (43, 59).

The analysis was constrained by the lack of multiple time points to allow a longitudinal analysis of virus dynamics and as such represents only a limited snapshot of the viral community. This study also cannot address the numerous RNA viruses that are present in NYC wastewater, and future studies would benefit from the inclusion of RNA to further understand the total viral community. While much work has focused on the vast diversity and abundance in ocean data sets (47, 49, 60), studies on urban wastewater have lagged behind. Sewage is an important urban ecosystem that can explain population-level attributes and provide a valuable resource for public health by providing insight into both the eukaryotic and bacterial viruses present in the population and environment (2). The results presented here offer insight into the phage communities across NYC, as well as their underlying potential functions and environments of origin. Understanding the biodiversity of wastewater treatment centers also can aid in making treatment processes more efficient by harnessing the innate ability of phages to target bacterial communities (61). Our study is a look into wastewater viral diversity and function and provides a deeper understanding of potential phage-host interactions in a complex environment.

## MATERIALS AND METHODS

### Sample data sets.

Metagenomic sequencing data from NYC sewage samples were collected by Maritz et al. (12). Data were downloaded from the NCBI Sequence Read Archive under BioProject no. PRJEB28033 with the following accession numbers: ERR2729796, ERR2729797, ERR2729798, ERR2729799, ERR2729800, ERR2729801, ERR2729802, ERR2729803, ERR2729804, ERR2729805, ERR2729806, ERR2729807, ERR2729808, ERR2729809, ERR2729810, and ERR2729811. The data consisted of samples from raw sewage collected across all NYC DEP wastewater treatment plants. Each sample includes raw sewage taken every 3 h over a 24-h period in November 2014. Approximately 1 ml of raw sewage was used for DNA extraction with the PowerSoil DNA isolation kit (Qiagen, catalog no. 12888). Sequencing libraries were constructed using the KAPA LTP library preparation kit (KAPA Biosystems, catalog no. KK8232) and sequenced on two lanes of a HiSeq Rapid Run with 2 × 250-bp paired-end chemistry, resulting in 10,751,683 raw paired-end reads.

Additional data came from wastewater samples collected in California, Illinois, Massachusetts, Pennsylvania, and Vermont with SRA accession numbers SRR5007225, SRR2062623, SRR5007352, SRR5007271, SRR2062049, SRR4236650, SRR4236649, SRR4236660, SRR4236648, SRR5007133, SRR4236663, SRR4244739, SRR5007150, SRR5007354, SRR4244858, SRR2060726, SRR5007116, SRR2062633, SRR5007313, SRR5007348, SRR4236662, SRR4236666, SRR5007146, SRR5007272, SRR4236664, and SRR8476230.

### Taxonomic assignment and assembly.

Viral taxonomic assignment was performed with VirMAP (1.0), which uses nucleotide and protein information to assign virus taxonomy (14). VirMAP was run using a quality filter set at Q15 and a kmer length of 20. Additionally, Illumina adapters were removed allowing 1 mismatch to determine the reads that would be processed. To perform metagenomic assembly, reads matching the human genome were first removed using Deconseq (0.4.3) using default parameters (62). The remaining reads from each sample were used as input for metaSpades, which was run with default parameters (63). Alpha and beta diversities were calculated using the “vegan” package in R (64).

### Virus prediction and annotation.

Assembled contigs were used as input for VirSorter (1.0.4) (17) and were run on Cyverse (65, 66) to identify putative viral contigs. Contigs from VirSorter categories 1 and 2 were selected for further analysis to minimize the chance of including nonviral sequences as contigs assigned to these 2 categories are the most likely to represent viral genomes. These contigs were mapped back to the Joint Genome Institute’s Integrated Microbial Genome/Virus database (accessed in November 2018) to identify ecosystems of origin, using a greedy approach to select for the best match (28). These contigs were also processed using the PHASTER API in February 2018 to detect prophages (39). Additionally, contigs were functionally annotated using HUMAnN2 (0.11.1) (32) and pfamscan (1.6) (67). Contigs were mapped to the UniRef50 database (1.1, downloaded from HUMAnN2 repository, November 2019) and further mapped to MetaCyc reactions and Gene Ontology (GO) terms using HUMAnN2 (0.11.1). MetaCyc reactions were plotted and analyzed with a cutoff value of 0.001. Open reading frames (ORFs) were predicted for each contig using Prodigal (2.6.3) (68) and used as input for pfamscan (1.6) to identify Pfams on each contig using HMMER (3.0) (33, 34). Heatmaps were generated using heatmaply (1.0.0) (69) in R and visualized as the square root of the relative abundance of each feature in each sample. Using the “vegan” package (2.5 to 6) in R, principal-coordinate analysis (PCoA) of Pfam domains was performed with Jaccard dissimilarity; comparisons of dissimilarities were defined using ANOVA.

### Virophage identification and analysis.

Virophage major capsid proteins (MCPs) were identified using both blastp (2.9.0) and hmmsearch (3.2.1) (34, 70). Contigs containing putative MCPs were examined for the presence of 3 other core virophage genes: a minor capsid gene, a cysteine protease gene, and a DNA-packaging gene. To generate an MCP phylogenetic tree, multiple alignments of complete and nearly complete MCPs from this study along with previously published MCPs were generated using Muscle (3.8.31) (71). The maximum-likelihood tree was constructed using FastTree (2.1.10) (Whelan Goldman model) (72) and visualized using iTOL (73).

### Phage-host prediction.

We predicted phage-host pairs by using Crass (2.1) (74) to detect CRISPR spacers and direct repeats. Spacers and repeats were mapped back to the assembled contigs, and those with a mismatch of <2 bp were retained for analysis. We clustered the spacers and repeats from each sample to determine their nucleotide similarity using CD-HIT (4.6.8) (75). The average cluster size for spacers was 1.1, indicating that most spacer sequences identified were unique. The average cluster size for direct repeats was 2.8, indicating that repeats can be identified more than once, as expected based on CRISPR-array architecture.

Contigs containing spacers and/or repeats were assigned taxonomy by alignment to the NCBI nt database (June 2019) using BLASTN (blast+ 2.9.0). Spacers were aligned to predicted viral contigs using BLASTN optimized for shorter sequences, as detailed in reference 76. For each viral contig, the bacterium with the best-matching CRISPR spacer was predicted as the host using a greedy approach (highest bit score, lowest E value, highest percent identity, longest length, and lowest number of mismatches and gaps).

### Gene-sharing network construction and clustering of viral contigs.

Viral clusters were identified using a shared gene content-based network analysis, where virus genomes and contigs are nodes in the network and sequence similarities are represented as edges, as described in Jang et al., 2019 (18). Briefly, for each sample, ORFs were predicted and compared all-to-all using BLASTP along with virus genomes in the viral RefSeq database (version 85, November 2017) and clustered using the Cyverse tool vConTACT2-Gene2Genome (1.1.0). The protein clusters were used as input to generate viral clusters using vConTACT2 (0.9.5) (18, 77). To identify viral contigs that were grouped with viral RefSeq genomes across the samples, output from vConTACT2 (0.9.5) for each sample was merged together to create a condensed network, visualized in Cytoscape (77).

### Bipartite network analysis.

Phage-bacterium network infection patterns were stored using an adjacency matrix with 285 columns representing VCs and 102 rows representing bacterial genera. BiMat (1.0) was used to calculate nestedness and modularity using the equiprobable null model and 10,000 iterations. Nestedness was tested using the nestedness temperature calculator (NTC) (78) and nestedness metric based on overlap and decreasing fill (NODF) (79). Modularity was tested using label propagation followed by bipartite recursively induced modularity (LP-BRIM) (80).

### Phage-host co-occurrence analysis.

A kmer=based taxonomic analysis was performed using Kaiju (1.7.2) (81) for the 16 NYC samples and 26 wastewater metagenomic samples collected in the United States and obtained from Integrated Microbial Genome (IMG) (82). Differential abundance between cities was performed using multinomial regression available from the songbird package (83). New York was chosen as the reference city. This analysis was run with 10,000 epochs and a batch size of 3 samples.

To estimate phage-microbe interactions, co-occurrence analysis was performed using mmvec (1.0) (84). From this, we were able to approximate the conditional probability of observing a specific microbe given that a specific phage was observed. This analysis was run with 1 latent dimension 500 epochs, a learning rate of 1e−5, and a batch size of 1,000 sequences; 4 samples were held out for cross validation.
