# Supplementary material for: Initial Mapping of the New York City Wastewater Virome
Source: mSystems. 2020 Jun 16;5(3):e00876-19. doi: 10.1128/mSystems.00876-19 (PMC7300365; doi:10.1128/mSystems.00876-19)
Supplement: TABLE S1 [file mSystems.00876-19-st001.pdf]

Table S1: Alpha diversity measurements

Calculated alpha diversity measurements (Simpson, Gini-Simpson, richness, Shannon’s H, and Pielou’s J) across samples.

ID	Sample	Simpson	Gini-Simpson	Richness	Shannon's H	Pielou's J
1	Brooklyn_1	0.16	0.84	176	0.61	0.12
2	BK_Q_MN	0.05	0.95	139	0.22	0.04
3	BK_Q	0.15	0.85	149	0.52	0.10
4	Brooklyn_2	0.08	0.92	131	0.33	0.07
5	Brooklyn_3	0.02	0.98	90	0.09	0.02
6	Staten Island_1	0.03	0.97	78	0.12	0.03
7	Staten Island_2	0.11	0.89	169	0.42	0.08
8	Brooklyn_4	0.13	0.87	199	0.49	0.09
9	Queens_1	0.20	0.80	480	0.84	0.14
10	Bronx	0.12	0.88	155	0.38	0.08
11	Queens_2	0.09	0.91	138	0.33	0.07
12	Queens_3	0.10	0.90	153	0.40	0.08
13	BX_MN	0.11	0.89	171	0.43	0.08
14	Manhattan	0.12	0.88	139	0.46	0.09
15	Queens_4	0.09	0.91	153	0.36	0.07
16	Queens_5	0.10	0.90	148	0.40	0.08

BK=Brooklyn; Q=Queens, MN=Manhattan; BX=Bronx
