# Supplementary figures and images for: Initial Mapping of the New York City Wastewater Virome
Source: mSystems. 2020 Jun 16;5(3):e00876-19. doi: 10.1128/mSystems.00876-19 (PMC7300365; doi:10.1128/mSystems.00876-19)

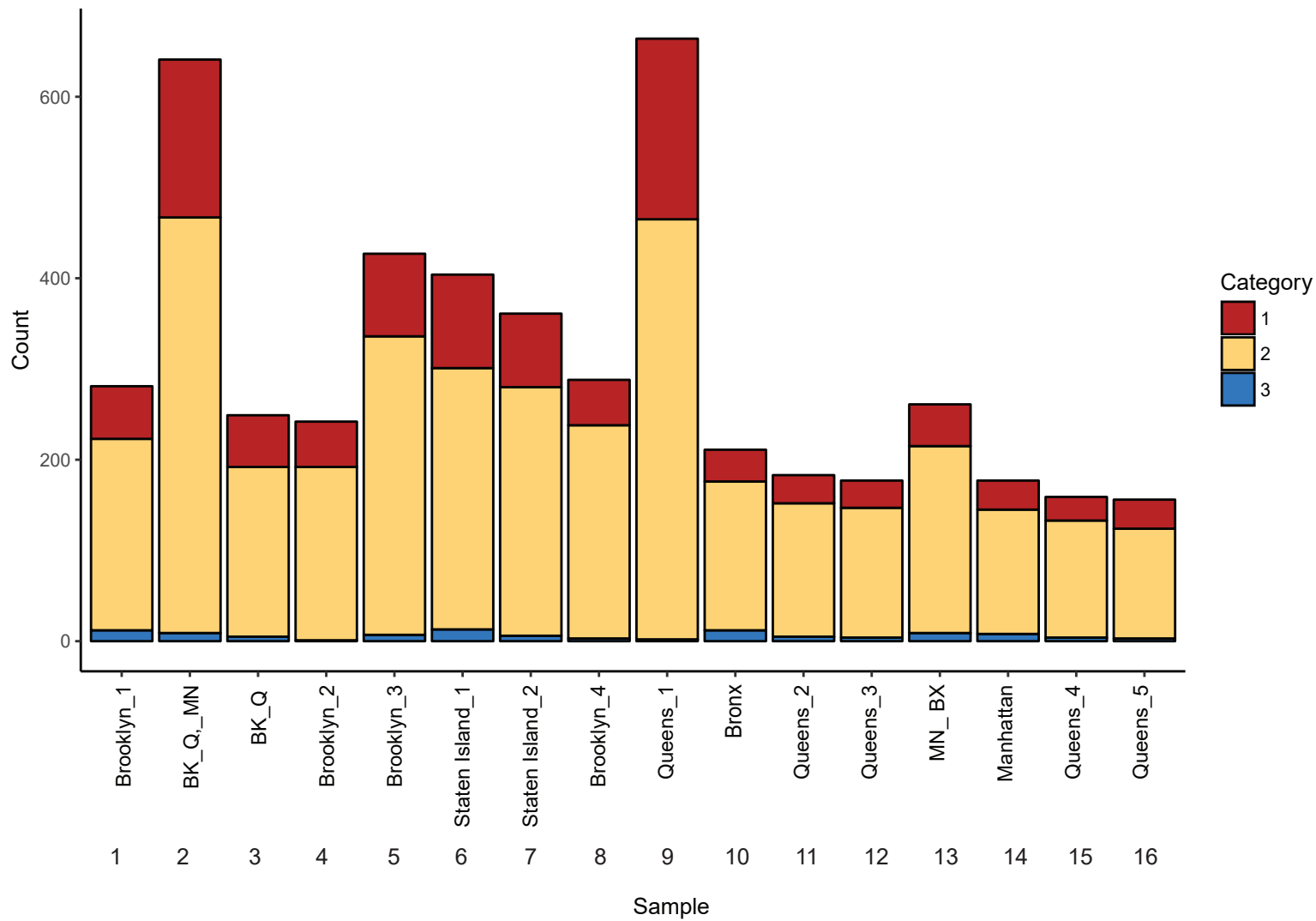

Supplement: FIG S1 [file mSystems.00876-19-sf001.pdf]

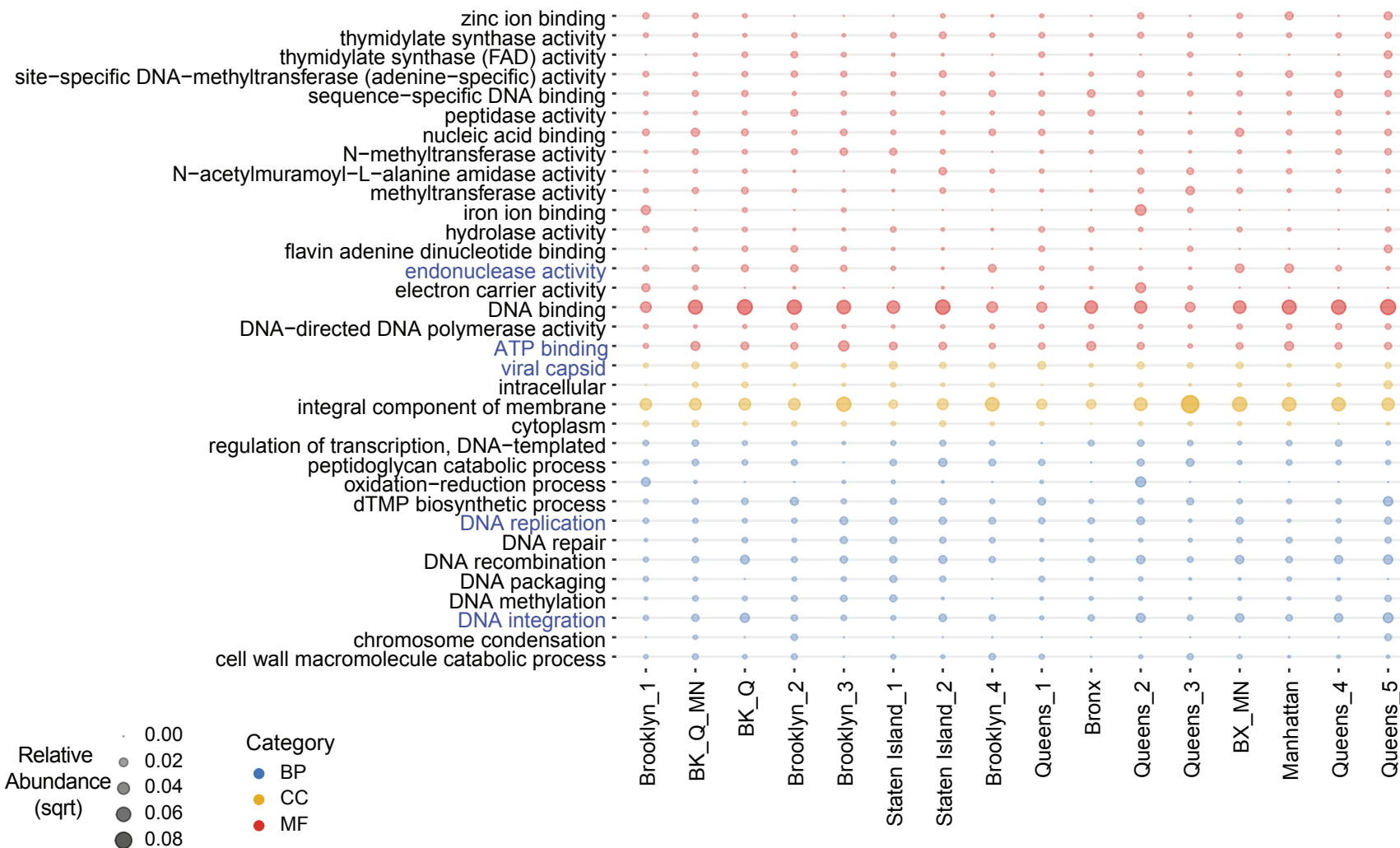

Supplement: FIG S2 [file mSystems.00876-19-sf002.pdf]

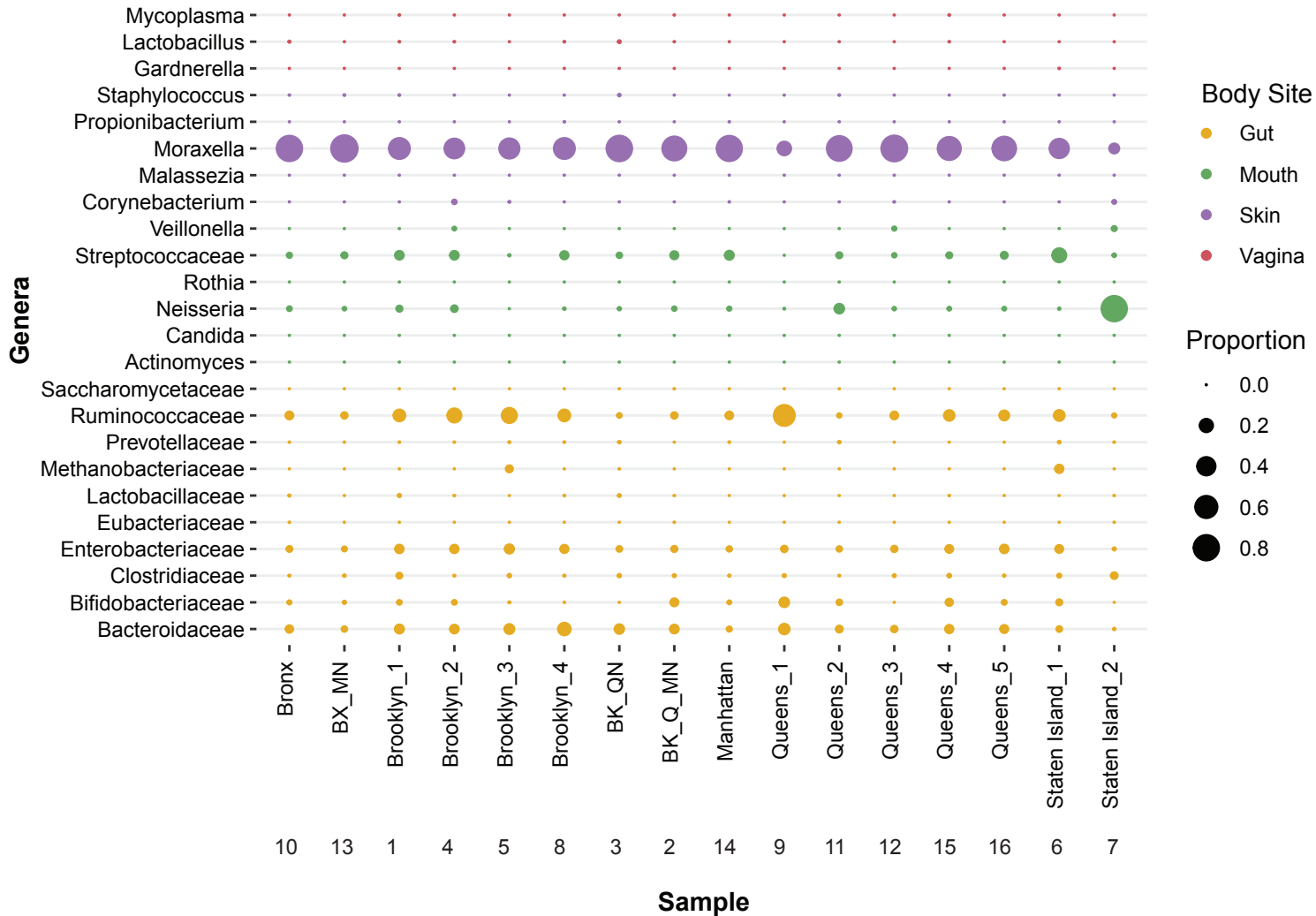

Supplement: FIG S4 [file mSystems.00876-19-sf004.pdf]

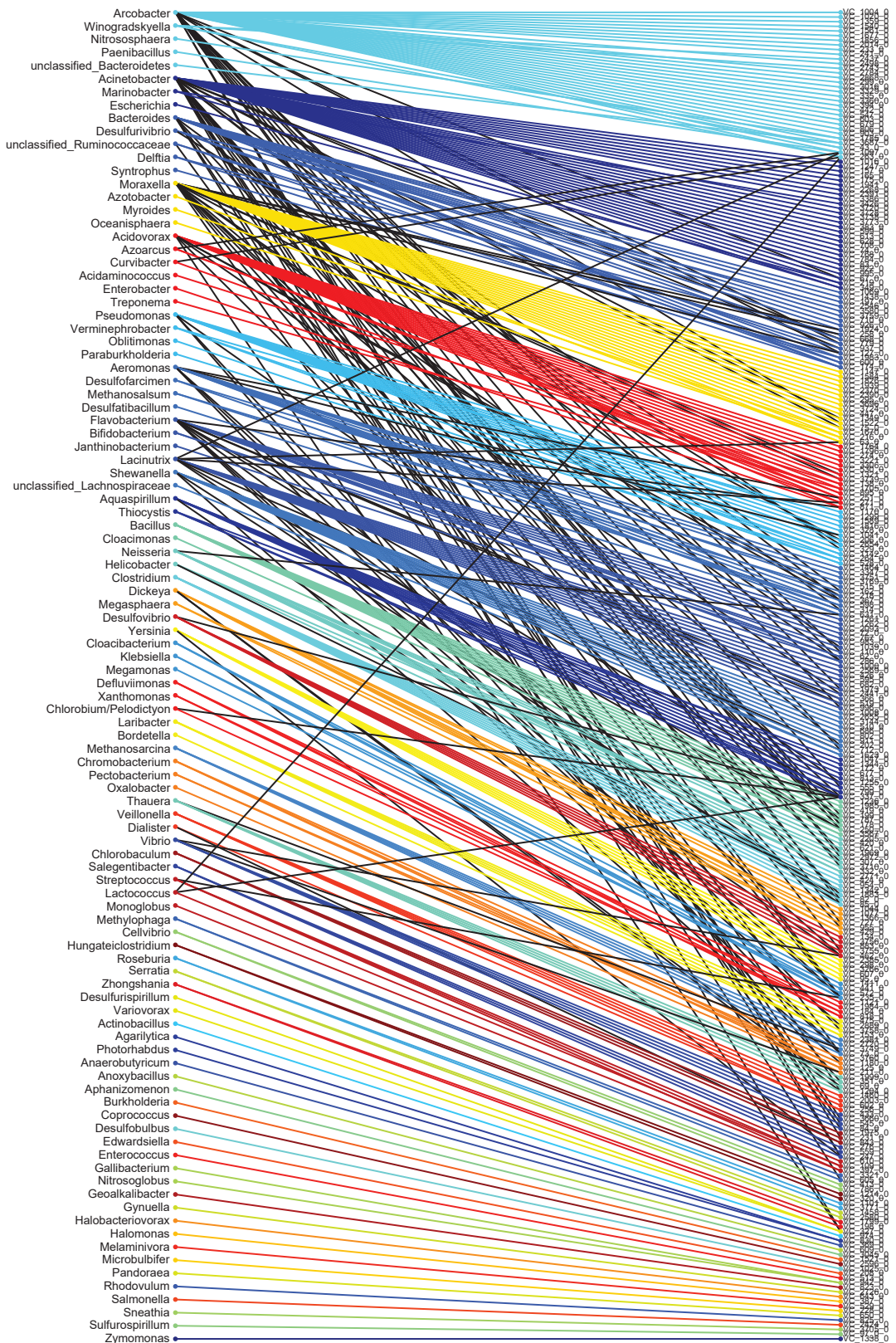

Supplement: FIG S5 [file mSystems.00876-19-sf005.pdf]
