# Supplementary material for: Initial Mapping of the New York City Wastewater Virome
Source: mSystems. 2020 Jun 16;5(3):e00876-19. doi: 10.1128/mSystems.00876-19 (PMC7300365; doi:10.1128/mSystems.00876-19)
Supplement: FIG S3 [file mSystems.00876-19-sf003.pdf]

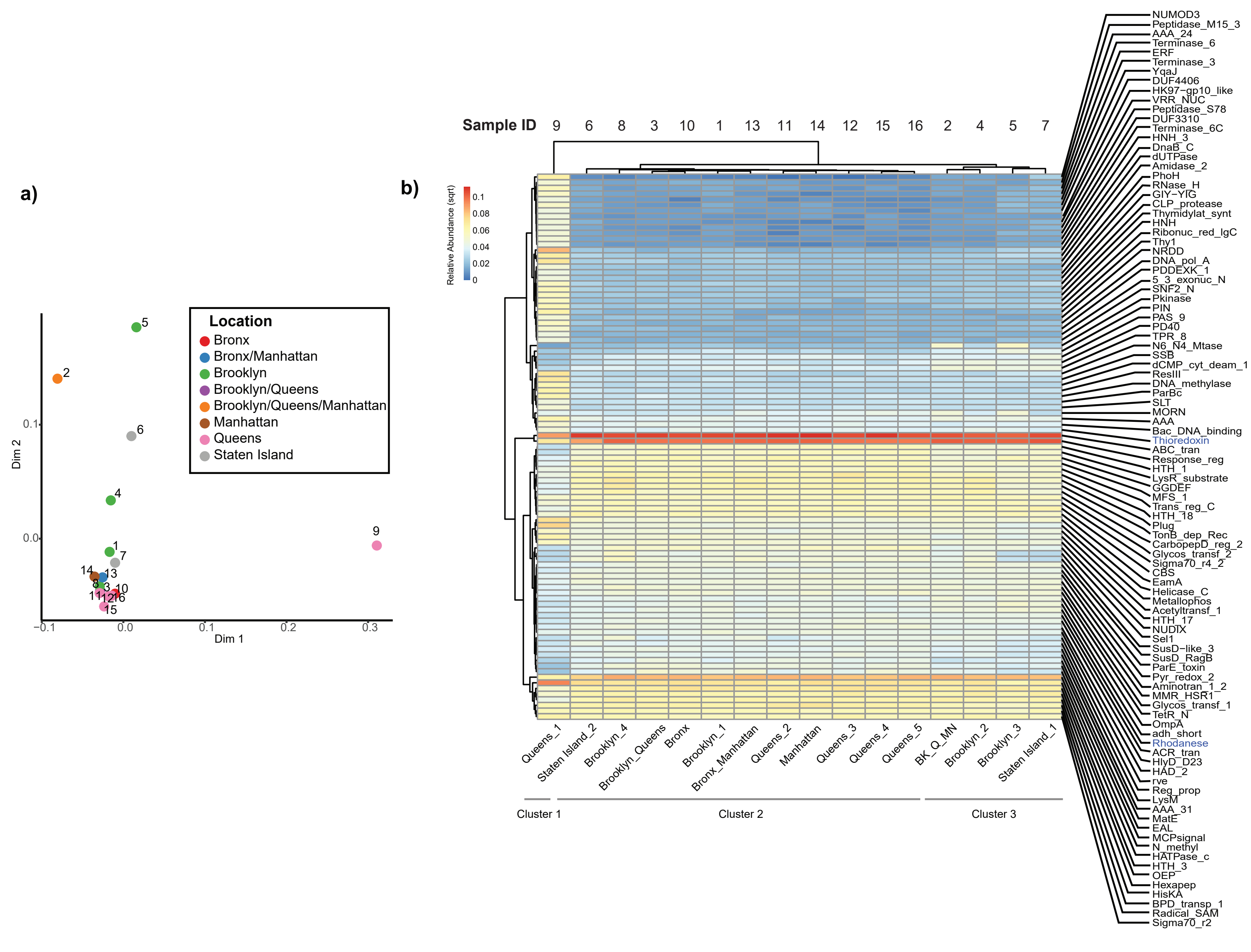

a)
Location
Bronx
Bronx/Manhattan
Brooklyn
Brooklyn/Queens
Brooklyn/Queens/Manhattan
Manhattan
Queens
Staten Island
Dim 2
Dim 1
0.1
0.0
−0.1
0.0
0.1
0.2
0.3
5
2
6
4
9
1
7
14
13
8
3
10
11
12
16
15
b)
Relative Abundance (sqrt)
0.1
0.08
0.06
0.04
0.02
0
Sample ID
9
6
8
3
10
1
13
11
14
12
15
16
2
4
5
7
Queens_1
Staten Island_2
Brooklyn_4
Brooklyn_Queens
Bronx
Brooklyn_1
Bronx_Manhattan
Queens_2
Manhattan
Queens_3
Queens_4
Queens_5
BK_Q_MN
Brooklyn_2
Brooklyn_3
Staten Island_1
Cluster 1
Cluster 2
Cluster 3
NUMOD3
Peptidase_M15_3
AAA_24
Terminase_6
ERF
Terminase_3
YqaJ
DUF4406
HK97−gp10_like
VRR_NUC
Peptidase_S78
DUF3310
Terminase_6C
HNH_3
DnaB_C
dUTPase
Amidase_2
PhoH
RNase_H
GIY−YIG
CLP_protease
Thymidylat_synt
HNH
Ribonuc_red_lgC
Thy1
NRDD
DNA_pol_A
PDDEXK_1
5_3_exonuc_N
SNF2_N
Pkinase
PIN
PAS_9
PD40
TPR_8
N6_N4_Mtase
SSB
dCMP_cyt_deam_1
ResIII
DNA_methylase
ParBc
SLT
MORN
AAA
Bac_DNA_binding
Thioredoxin
ABC_tran
Response_reg
HTH_1
LysR_substrate
GGDEF
MFS_1
Trans_reg_C
HTH_18
Plug
TonB_dep_Rec
CarbopepD_reg_2
Glycos_transf_2
Sigma70_r4_2
CBS
EamA
Helicase_C
Metallophos
Acetyltransf_1
HTH_17
NUDIX
Sel1
SusD−like_3
SusD_RagB
ParE_toxin
Pyr_redox_2
Aminotran_1_2
MMR_HSR1
Glycos_transf_1
TetR_N
OmpA
adh_short
Rhodanese
ACR_tran
HlyD_D23
HAD_2
rve
Reg_prop
LysM
AAA_31
MatE
EAL
MCPsignal
N_methyl
HATPase_c
HTH_3
OEP
Hexapep
HisKA
BPD_transp_1
Radical_SAM
Sigma70_r2
