# Supplementary material for: Initial Mapping of the New York City Wastewater Virome
Source: mSystems. 2020 Jun 16;5(3):e00876-19. doi: 10.1128/mSystems.00876-19 (PMC7300365; doi:10.1128/mSystems.00876-19)
Supplement: TABLE S2 [file mSystems.00876-19-st002.pdf]

Table S2 : Core protein families

These 31 protein families were found to be present in at least 12/16 samples at a relative abundance of 0.25% or higher.

Family	Pfam ID	Function
ABC_tran	PF00005	ABC transporter
BPD_transp_1	PF00528	Binding-protein-dependent transport system inner membrane component
CarbopepD_reg_2	PF13715	CarboxypepD reg-like domain
CBS	PF00571	CBS domain
EamA	PF00892	EamA-like transporter family
GGDEF	PF00990	Diguanylate cyclase, GGDEF domain
Glycos_transf_2	PF00535	Glycosyl transferase family 2
HATPase_c	PF02518	Histidine kinase-, DNA gyrase B-, and HSP90-like ATPase
Helicase_C	PF00271	Helicase conserved C-terminal domain
Hexapep	PF00132	Bacterial transferase hexapeptide
HisKA	PF00512	His Kinase A (phospho-acceptor) domain
HTH_1	PF00126	Bacterial regulatory helix-turn-helix protein, lysR family
HTH_17	PF12728	Helix-turn-helix domain
HTH_18	PF12833	Helix-turn-helix domain
HTH_3	PF01381	Helix-turn-helix domain
LysR_substrate	PF03466	LysR substrate binding domain
MFS_1	PF07690	Major Facilitator Superfamily
N_methyl	PF07963	Prokaryotic N-terminal methylation motif
NUDIX	PF00293	NUDIX domain
OEP	PF02321	Outer membrane efflux protein
Pkinase	PF00069	Protein kinase domain
Plug	PF07715	TonB-dependent Receptor Plug Domain
Radical_SAM	PF04055	Radical SAM superfamily
Response_reg	PF00072	Response regulator receiver domain
Sel1	PF08238	Sel1 repeat
Sigma70_r2	PF04542	Sigma-70 region 2
Sigma70_r4_2	PF08281	Sigma-70, region 4
SusD_RagB	PF07980	SusD family
TetR_N	PF00440	Bacterial regulatory proteins, tetR family
TonB_dep_Rec	PF00593	TonB dependent receptor
Trans_reg_C	PF00486	Transcriptional regulatory protein, C terminal
