# Supplementary material for: Initial Mapping of the New York City Wastewater Virome
Source: mSystems. 2020 Jun 16;5(3):e00876-19. doi: 10.1128/mSystems.00876-19 (PMC7300365; doi:10.1128/mSystems.00876-19)
Supplement: TABLE S3 [file mSystems.00876-19-st003.pdf]

Table S3: CRISPR array identification and clustering

Total number of CRISPR spacers and direct repeats (DR) identified and mapped back to contigs and clustering statistics calculated using CD-HIT.

ID	SAMPLE NAME	TOTAL IDENTIFIED SPACERS	TOTAL MAPPED SPACERS	TOTAL SPACER CLUSTERS	AVERAGE SPACER CLUSTER SIZE	TOTAL IDENTIFIED DRs	TOTAL MAPPED DRs	TOTAL DR CLUSTERS	AVERAGE DR CLUSTER SIZE
1	Brooklyn_1	20,168	9,976	18,807	1.07	1,957	599	619	3.16
2	BK_Q_MN	38,687	19,223	35,565	1.09	2,929	895	905	3.24
3	BK_Q	21,892	10,839	20,062	1.09	1,541	529	541	2.85
4	Brooklyn_2	21,095	10,008	19,637	1.07	1,640	604	638	2.57
5	Brooklyn_3	14,642	7,097	13,715	1.07	1,664	601	678	2.45
6	Staten Island_1	18,724	9,093	17,251	1.09	1,886	638	667	2.83
7	Staten Island_2	22,826	11,216	21,191	1.08	1,870	610	641	2.92
8	Brooklyn_4	21,888	10,854	20,154	1.09	1,964	607	565	3.48
9	Queens_1	5,809	3,217	5,447	1.07	897	377	468	1.92
10	Bronx	22,447	10,968	20,663	1.09	1,304	458	482	2.71
11	Queens_2	22,996	11,127	21,169	1.09	1,737	555	593	2.93
12	Queens_3	17,979	8,374	16,825	1.07	1,359	484	560	2.43
13	BX_MN	23,273	11,636	21,504	1.08	1,452	493	517	2.81
14	Manhattan_1	24,087	11,280	22,355	1.08	1,891	630	681	2.78
15	Queens_4	19,241	8,880	18,146	1.06	1,618	518	537	3.01
16	Queens_5	16,016	7,559	14,986	1.07	1,268	455	462	2.74

BK=Brooklyn; Q=Queens, MN=Manhattan; BX=Bronx
