# Supplementary material for: Initial Mapping of the New York City Wastewater Virome
Source: mSystems. 2020 Jun 16;5(3):e00876-19. doi: 10.1128/mSystems.00876-19 (PMC7300365; doi:10.1128/mSystems.00876-19)
Supplement: FIG S6 [file mSystems.00876-19-sf006.pdf]

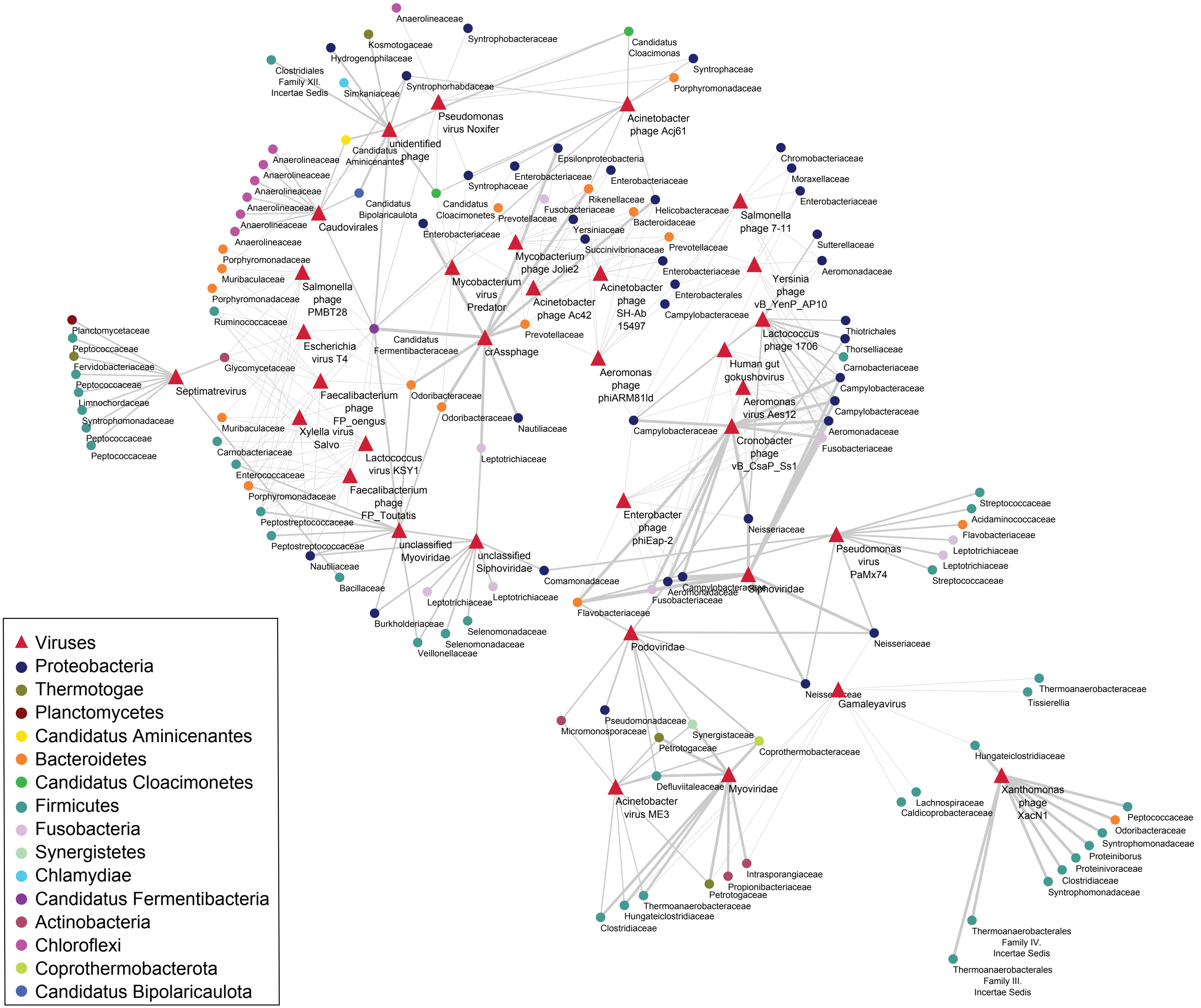

Anaerolineaceae
Syntrophobacteraceae
Kosmotogaceae
Hydrogenophilaceae
Candidatus Cloacimonas
Syntrophaceae
Clostridiales Family XII. Incertae Sedis
Simkaniaceae
Syntrophorhabdaceae
Porphyromonadaceae
Pseudomonas virus Noxifer
Acinetobacter phage Acj61
unidentified phage
Candidatus Aminicenantes
Anaerolineaceae
Anaerolineaceae
Anaerolineaceae
Anaerolineaceae
Anaerolineaceae
Anaerolineaceae
Caudovirales
Candidatus Bipolaricaulota
Epsilonproteobacteria
Enterobacteriaceae
Syntrophaceae
Enterobacteriaceae
Chromobacteriaceae
Moraxellaceae
Enterobacteriaceae
Candidatus Cloacimonetes
Prevotellaceae
Fusobacteriaceae
Rikenellaceae
Helicobacteraceae
Bacteroidaceae
Salmonella phage 7-11
Enterobacteriaceae
Yersiniaceae
Succinivibrionaceae
Prevotellaceae
Sutterellaceae
Porphyromonadaceae
Muribaculaceae
Porphyromonadaceae
Ruminococcaceae
Salmonella phage PMBT28
Mycobacterium phage Jolie2
Mycobacterium virus Predator
Enterobacteriaceae
Aeromonadaceae
Acinetobacter phage Ac42
Acinetobacter phage SH-Ab 15497
Enterobacterales
Campylobacteraceae
Yersinia phage vB_YenP_AP10
Planctomycetaceae
Peptococcaceae
Fervidobacteriaceae
Peptococcaceae
Limnochordaceae
Syntrophomonadaceae
Peptococcaceae
Peptococcaceae
Septimatrevirus
Glycomycetaceae
Escherichia virus T4
Candidatus Fermentibacteraceae
crAssphage
Prevotellaceae
Thiotrichales
Lactococcus phage 1706
Thorselliaceae
Carnobacteriaceae
Aeromonas phage phiARM81ld
Human gut gokushovirus
Campylobacteraceae
Aeromonas virus Aes12
Campylobacteraceae
Faecalibacterium phage FP_oengus
Odoribacteraceae
Odoribacteraceae
Nautiliaceae
Muribaculaceae
Xylella virus Salvo
Campylobacteraceae
Aeromonadaceae
Cronobacter phage vB_CsaP_Ss1
Fusobacteriaceae
Carnobacteriaceae
Lactococcus virus KSY1
Leptotrichiaceae
Enterococcaceae
Faecalibacterium phage FP_Toutatis
Porphyromonadaceae
Peptostreptococcaceae
Peptostreptococcaceae
Enterobacter phage phiEap-2
Neisseriaceae
Streptococcaceae
Acidaminococcaceae
Flavobacteriaceae
Leptotrichiaceae
Leptotrichiaceae
Streptococcaceae
Pseudomonas virus PaMx74
unclassified Myoviridae
unclassified Siphoviridae
Nautiliaceae
Bacillaceae
Comamonadaceae
Campylobacteraceae
Aeromonadaceae
Fusobacteriaceae
Siphoviridae
Leptotrichiaceae
Leptotrichiaceae
Flavobacteriaceae
Burkholderiaceae
Selenomonadaceae
Selenomonadaceae
Veillonellaceae
Podoviridae
Neisseriaceae
Neisseriaceae
Gamaleyavirus
Thermoanaerobacteraceae
Tissierella
Pseudomonadaceae
Micromonosporaceae
Synergistaceae
Petrotogaceae
Coprothermobacteraceae
Hungateiclostridiaceae
Defluviitaleaceae
Acinetobacter virus ME3
Myoviridae
Lachnospiraceae
Caldicoprobacteraceae
Xanthomonas phage XacN1
Peptococcaceae
Odoribacteraceae
Syntrophomonadaceae
Proteiniborus
Proteinivoraceae
Clostridiaceae
Syntrophomonadaceae
Intrasporangiaceae
Propionibacteriaceae
Petrotogaceae
Thermoanaerobacteraceae
Hungateiclostridiaceae
Clostridiaceae
Thermoanaerobacterales Family IV. Incertae Sedis
Thermoanaerobacterales Family III. Incertae Sedis
Viruses
Proteobacteria
Thermotogae
Planctomycetes
Candidatus Aminicenantes
Bacteroidetes
Candidatus Cloacimonetes
Firmicutes
Fusobacteria
Synergistetes
Chlamydiae
Candidatus Fermentibacteria
Actinobacteria
Chloroflexi
Coprothermobacterota
Candidatus Bipolaricaulota
